# An investigation of pyrexia of unknown origin in Shamva District, Zimbabwe, September 2015

**DOI:** 10.11604/pamj.supp.2018.30.1.15270

**Published:** 2018-05-18

**Authors:** Daniel Chirundu, Tsitsi Juru, Nsiande Lema, Rayyan Muhammad Garba, Joseph Asamoah Frimpong

**Affiliations:** 1 Kadoma City Health Department, Kadoma, Zimbabwe; 2 Zimbabwe Field Epidemiology Training Program, Harare, Zimbabwe; 3 Tanzania Field Epidemiology Training program, Dar es Salaam, Tanzania; 4 Nigeria Field Epidemiology Training program, Abuja, Nigeria; 5 African Field Epidemiology Network, Accra, Ghana

**Keywords:** Outbreak investigation, pyrexia, Zimbabwe

## Abstract

Outbreak investigation is a key component of public health training. A good outbreak investigation can go beyond determining the causative agent by recommending policies to be formulated by policy makers. This case study simulates a real-life investigation of pyrexia of unknown origin in Shamva District, Zimbabwe, during the period of September to October 2015. It aims at reinforcing principles and skills taught in class on outbreak investigation, study design and policy initiation. The target audience for the case study is Field Epidemiologists at their advanced level of training. It is expected to be completed in approximately 2 hours.

General instructions: ideally, 1 to 2 facilitator(s) is/are required to facilitate the case study for 10 to 20 participants. The facilitator should request participants to read a paragraph out loud, going around the room to give each participant a chance to read. When the participant reads a question, the facilitator encourages all participants to engage in discussions, perform calculations, and draw graphs among other tasks. The facilitators request the participants to play different roles or take different sides in answering a question. As a result, participants learn from each other, not just from the facilitators.

## How to use this Study

**Audience:** Field Epidemiologist and other person(s) interested in the case study.

**Pre-requisite:** before using this case study, case study participants should have received lectures or other instruction in outbreak investigation, use of Epi info 7 visual dashboard to run aberration algorithm, study design, measures of association, and policy formulation and analysis.

**Materials needed:** flipchart or whiteboard with markers.

**Level of training:** advanced outbreak investigation.

**Time required:** approximately 2 hours

**Language:** English

## Competing interest

The authors declare no competing interest.
